# Early-Onset Sepsis as an Early Predictor for Retinopathy of Prematurity: A Meta-analysis

**DOI:** 10.1055/a-2369-6690

**Published:** 2024-08-13

**Authors:** Salma El Emrani, Lotte E. van der Meeren, Esther J.S. Jansen, Jelle J. Goeman, Jacqueline U.M. Termote, Enrico Lopriore, Nicoline E. Schalij-Delfos

**Affiliations:** 1 Department of Ophthalmology, Leiden University Medical Center, Leiden, The Netherlands; 2 Neonatology, Department of Pediatrics, Willem-Alexander Children's Hospital, Leiden University Medical Center, Leiden, The Netherlands; 3 Department of Pathology, Leiden University Medical Center, Leiden, The Netherlands; 4 Department of Pathology, Erasmus Medical Center, Rotterdam, The Netherlands; 5 Neonatology, Department of Women and Neonate, Wilhelmina Children's Hospital, University Medical Center Utrecht, Utrecht, The Netherlands; 6 Medical Statistics, Department of Biomedical Data Science, Leiden University Medical Center, Leiden, The Netherlands

**Keywords:** retinopathy of prematurity, early-onset sepsis, prematurity, meta-analysis

## Abstract

**Objective** Neonatal sepsis has been established as a risk factor for retinopathy of prematurity (ROP) but previous meta-analyses have predominately focused on late-onset sepsis (LOS). This meta-analysis aims to explore the association between early-onset sepsis (EOS) and the risk of ROP.

**Study Design** Observational studies reporting (unadjusted) data on proven EOS in neonates with ROP were included. PubMed, Embase, and Cochrane Library were searched. Proven EOS was defined as a positive blood or cerebrospinal fluid culture. Effect sizes were calculated by using logistic random-effects models and meta-regression analyses. Primary outcomes were any stage ROP and severe ROP (≥stage 3, type I, aggressive [posterior] ROP, plus disease or requiring treatment). Potential confounders explored were gestational age at birth, birth weight, small for gestational age, maternal steroid use, necrotizing enterocolitis, LOS, and mechanical ventilation duration.

**Results** Seventeen studies reporting the incidence of proven EOS in neonates with ROP were included. Proven EOS showed no significant association with any stage ROP (odds ratio [OR] = 1.90; 95% confidence interval [CI]: 0.96–3.79, *p* = 0.067) but heterogeneity between studies was significantly high. Neonates with proven EOS had an increased risk for severe ROP (OR = 2.21; 95% CI: 1.68–2.90), and no significant confounders influencing this effect size were found in the meta-regression analysis.

**Conclusion** Neonates with proven EOS are at increased risk of severe ROP. Neonatologists need to be aware that EOS is an early predictor of ROP and should adapt their policy and treatment decisions where possible to reduce ROP.

**Key Points**

This meta-analysis reveals a 2.2-fold increased risk of severe ROP in neonates with proven EOS.

Future studies should distinguish between EOS and LOS when investigating risk factors of ROP.

Treatment decisions should be adapted where possible in neonates with EOS before ROP screening begins.

Retinopathy of prematurity (ROP) is a multifactorial retinal disorder affecting 10 to 25% of neonates born under 32 weeks of gestation and continues to be the leading cause of visual impairment and childhood blindness worldwide. 1 2 Even though the majority of ROP cases are mild and usually self-limiting, advances in neonatal care and lowering the age limit of neonatal intensive care treatment have led to increased survival of extremely premature infants at risk of (severe) ROP. 3 4 Hence, accurate screening and timely detection and treatment of ROP are crucial in preventing progression to severe visual impairment and blindness.

Neonatal sepsis is an independent risk factor for ROP and is therefore included in the Dutch ROP screening criteria. 5 6 The mechanism behind this association is believed to be due to cytokine and angiogenesis factor production. 7 8 Sepsis releases inflammatory mediators that increase hypoxia-inducible factor (HIF-1α) concentrations, which may exacerbate phase II of ROP. 9 10 Additionally, sepsis induces an oxidative stress response that elevates vascular endothelial growth factor-2 (VEGF-2) levels, which may also initiate phase II of ROP. 11 12 Furthermore, perinatal infection can lead to decreased insulin-like growth factor-1 (IGF-1) levels, which promotes phase I of ROP. 13

The onset of sepsis may be a valuable tool in understanding the pathophysiology behind the first phase of ROP. Early-onset sepsis (EOS) is acquired through vertical transmission in the first 72 hours of life, while late-onset sepsis (LOS) is acquired through hospital and community environmental microorganisms beyond the first 72 hours of life and occurs more often. 14 15 Since studies have shown that placental inflammation/infection through vertical transmission leads to an increased risk of ROP, it may be beneficial to investigate the independent effect of EOS on ROP. 16 17

To date, meta-analyses on the association between sepsis and ROP have predominately focused on LOS and not on EOS. Extra awareness for ROP risk factors shortly after birth can provide the opportunity to tailor neonatal treatment decisions in phase I of ROP well before the first ROP screening is performed. Hence, we performed a systematic review and meta-analysis to investigate the association between EOS and the risk of ROP development to fill in the knowledge gap and potentially identify high-risk infants in an earlier stage.

## Materials and Methods

### Sources

This systematic review was conducted according to PRISMA (Preferred Reporting Items for Systematic Reviews and Meta-analyses) guidelines and is registered in PROSPERO (identifier: CRD42022380411; Supplementary Material S1 [available in the online version]). 18 The online electronic databases PubMed, Embase, and Cochrane Library were searched up until February 2024 using combinations of the following keywords: “Placenta,” “Sepsis,” “Risk Factors,” AND “Retinopathy of Prematurity.” Additionally, various synonyms were added as Medical Subject Headings (MESH) terms and free-text words.

### Study Selection

Eligibility was assessed through title and abstract screening with subsequent full-text evaluation when included. Studies were eligible for inclusion when (unadjusted) data were reported on the association between EOS and ROP. Studies were excluded when no distinction was made between EOS and LOS and when EOS was diagnosed based on clinical symptoms alone. Further exclusion criteria were case reports, case series, reviews, editorials, unavailable full text, animal studies, and when the study population consisted entirely of multiple pregnancies due to its confounding effect. 19 Eligibility was independently identified by three reviewers (S.E., L.E.M., and N.E.S-D.) and discrepancies were resolved through discussion.

The primary outcomes were any stage ROP and severe ROP (i.e., ≥stage 3, type I, aggressive [posterior] ROP, plus disease or requiring treatment). Due to their known potential confounding effect on ROP, the following clinical data were explored: gestational age (GA) at birth (weeks), birth weight (BW; g), small for gestational age (SGA; BW <10th centile), maternal steroid use, necrotizing enterocolitis (any stage), LOS (suspected/proven), and mechanical ventilation (days). 5 Proven EOS was defined as positive blood or cerebrospinal fluid culture in the first 72 hours after birth. 20 Neonates with EOS were compared with neonates without EOS for both any stage and severe ROP. Potential confounders were compared between any stage ROP and no ROP, and between severe ROP and no/mild ROP.

### Quality Assessment

The quality assessment was performed using the Newcastle–Ottawa Scale for case–control and cohort studies. 21 Three study aspects were assessed: selection (0–4 points), comparability (0–2 points), and exposure/outcome (0–3 points). Scoring was based on the association between EOS and ROP as the primary outcome and comparability scoring was based on adjustment for GA and an additional potential confounder reported in our study.

### Statistical Analysis

Statistical analyses were performed using RStudio, version 4.2.1 (RStudio, PBC, Boston, MA) with the metafor package and were assisted by a statistician (J.J.G). 22 Data are presented using *n* / *N* (%) and odds ratio (OR) with 95% confidence interval (CI). Reported unadjusted 2 × 2 table data were used to recalculate ORs and 95% CIs per study. These data were combined by using logistic random-effects models due to anticipated heterogeneity. Q-statistics were calculated to evaluate heterogeneity between studies, and *I* ^2^ -statistics were calculated to observe total variation between studies due to heterogeneity beyond chance. *p* -Values <0.05 were determined as statistically significant. Publication bias was estimated through funnel plots, failsafe numbers, and trim-and-fill functions.

To explain observed heterogeneity, the mean/median difference for continuous variables and incidence (%) difference for dichotomous variables were calculated between any stage and no ROP, and severe and no/mild ROP. To include more studies ( *N* ) in the meta-regression analysis, no distinction was made between median and mean for continuous variables. Univariate meta-regression models were performed to identify variables that potentially influence the effect size of proven EOS on ROP. The Bonferroni correction for multiple comparisons was used to account for multiple testing, in which a *p* -value of 0.05/number of included variables was considered statistically significant.

Subsequently, multivariate meta-regression analysis was performed using significant variables found in univariate analysis. Since GA and BW are highly correlated and adjusting for both variables in multivariate analysis will cause multicollinearity, only one variable should be included in multivariate analysis when both variables are significant, preferably with SGA. Data are presented using β-coefficient with 95% CI, which signifies a higher association between proven EOS and ROP in studies with large between-group differences in potential confounders when positive and lower when negative.

## Results

**Fig. 1:**
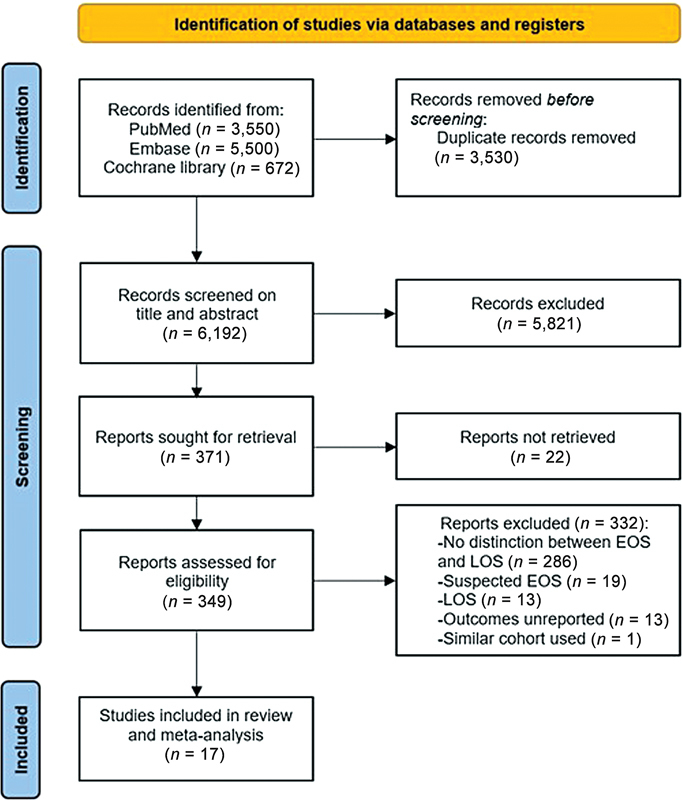
PRISMA flowchart of study inclusion. EOS, early-onset sepsis; LOS, late-onset sepsis. PRISMA, Preferred Reporting Items for Systematic Reviews and Meta-analyses.

The search strategy yielded 9,722 articles. After excluding duplicates, 6,192 titles and abstracts were screened. A total of 5,821 articles were excluded after a primary assessment based on inclusion and exclusion criteria. After a full-text assessment of the remaining 371 articles, 17 studies were included ( Fig. 1 ).

### Quality Analysis

Quality assessment is shown in Table 1 . Ten studies were categorized as high quality (8–9 points), while the remaining seven studies received moderate quality scores (6–7 points). Points were mainly lost due to the absence of confounder adjustment or the presence of multicollinearity due to adjusting for both GA and BW. The revisited International Classification of ROP was used for ROP classification in all included studies. 23

**Table 1:** Quality assessment of included studies

First author (y)	Selection (max 4 points)	Comparability (max 2 points)	Outcome (max 3 points)
Klinger, 2010 34	4	2	3
Chen, 2011 24	4	1	3
Silveira, 2011 25	4	0	3
Jimenez, 2012 26	4	1	3
Mularoni, 2014 35	4	1	2
Fonseca, 2018 27	4	2	3
Goldstein, 2019 36	4	1	3
Jiang, 2019 28	4	1	3
Celik, 2021 37	4	0	3
Nordberg, 2021 29	4	0	3
Abdel Salam Gomaa, 2021 38	4	0	3
Carranza-Mendizabal, 2021 30	4	1	3
Bonafiglia, 2022 31	4	1	3
Gudu, 2022 32	4	0	3
Duggan, 2023 39	4	2	3
Dincer, 2023 40	4	0	3
Boo, 2024 33	4	2	3

### Early-Onset Sepsis and Any Stage Retinopathy of Prematurity

Ten studies assessed the presence of proven EOS in neonates with any stage ROP compared with neonates without ROP, with sample sizes ranging from 74 to 14,008 ( Supplementary Table S1 [available in the online version]). 24 25 26 27 28 29 30 31 32 33

**Fig. 2:**
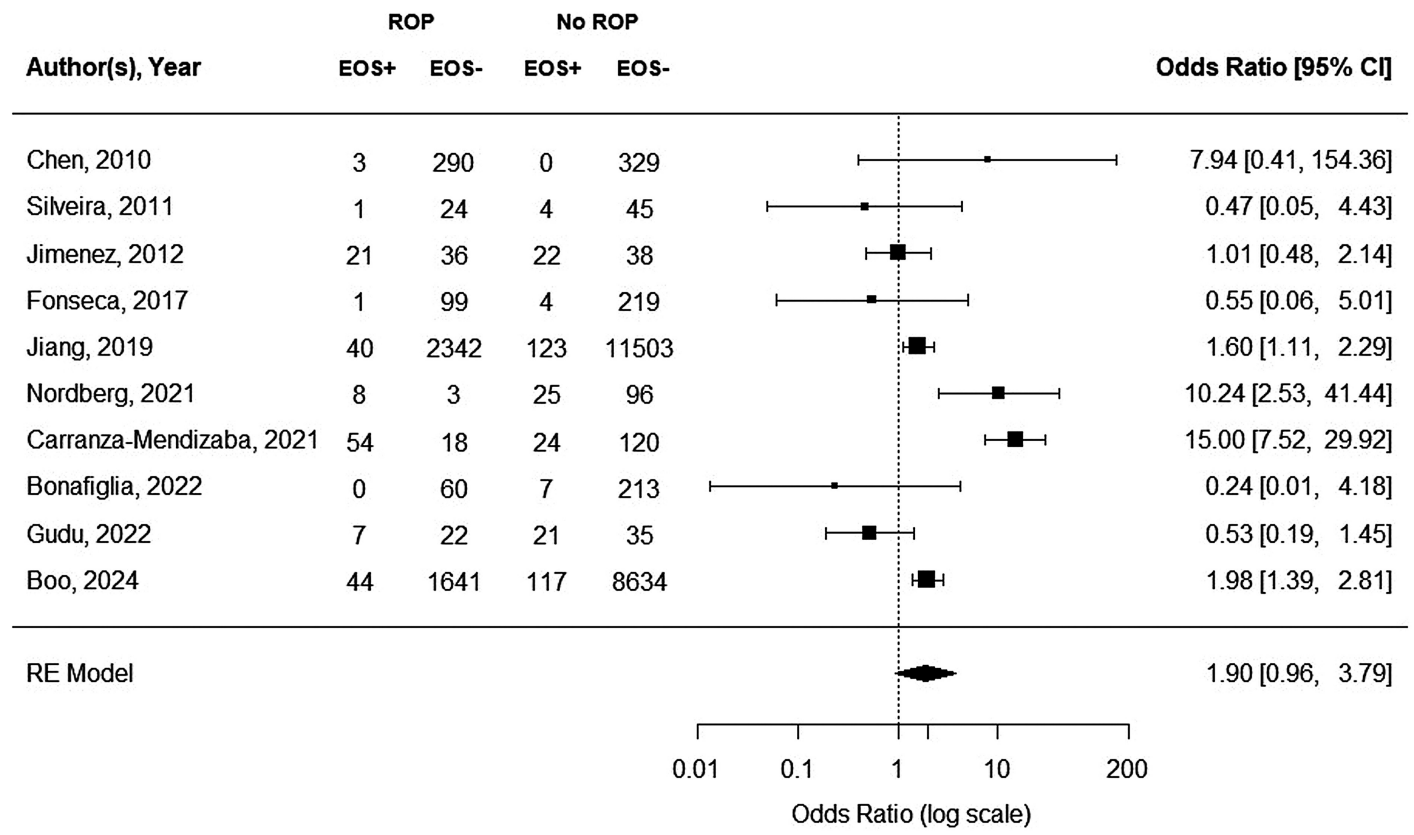
Forest plot of the association between proven EOS and any stage ROP. Heterogeneity: Q = 55.2; *I* ^2^ = 83.7%; *p* < 0.0001. 95% CI, 95% confidence interval; EOS, early-onset sepsis; ROP, retinopathy of prematurity.

Proven EOS showed no association with any stage ROP (OR = 1.90; 95% CI: 0.96–3.79, *p* = 0.0665; Fig. 2 ). Significantly high heterogeneity was observed (Q = 55.2, *I* ^2^ = 83.7%, *p* <0.0001). Neither visual inspection and trim-and-fill number ( *n* = 0) of the funnel plot, nor the failsafe number ( *n* = 77, Kendall'sτ = − 0.02, *p* = 1.0) showed indication of publication bias ( Supplementary Fig. S1 [available in the online version]).

Secondary analyses were performed to explore the found heterogeneity. Univariate meta-regression analysis was used to analyze the possible influence of these variables as moderators on the effect size of proven EOS on any stage ROP ( Table 2 ). Two factors were found to be significant moderators (number of studies: 3–8): GA difference (β-coefficient = 1.5; 95% CI: 0.6–2.3, *p* = 0.0005) and mechanical ventilation duration difference (β-coefficient −0.4; 95% CI: −0.6 to −0.2, *p* = 0.0002) between any stage ROP group and no ROP group. Multivariate meta-regression analysis was not possible due to a low number of studies reporting both GA difference and mechanical ventilation duration difference ( *N* < 4).

**Table 2:** Meta-regression regarding the influence of potential confounders on early-onset sepsis and any stage retinopathy of prematurity

Univariate	*N*	Estimate	SE	Z-value	*p* -Value	CI lower	CI higher	*R*^2^ (%)	*I*^2^ (%)
Gestational age at birth	7	1.4519	0.4199	3.4577	0.0005	0.6289	2.2749	84.99	42.21
Birth weight	7	0.0069	0.0076	0.9085	0.3636	−0.0080	0.0218	0.00	86.29
Small for gestational age	3	0.1313	0.0780	1.6834	0.0923	−0.0216	0.02841	100.00	0.00
Maternal steroids	7	0.0236	0.0418	0.5646	0.5723	−0.0583	0.1054	22.02	81.53
Necrotizing enterocolitis	7	−0.1196	0.0772	−1.5494	0.1213	−0.2708	0.0317	8.11	83.76
Late-onset sepsis	8	−0.0403	0.0306	−1.3179	0.1875	−0.1002	0.0196	0.00	84.27
Mechanical ventilation	3	−0.3959	0.1046	−3.7854	0.0002	−0.6009	−0.1909	100.00	0.00

Abbreviation: CI, confidence interval.

### Early-Onset Sepsis and Severe Retinopathy of Prematurity

**Fig. 3:**
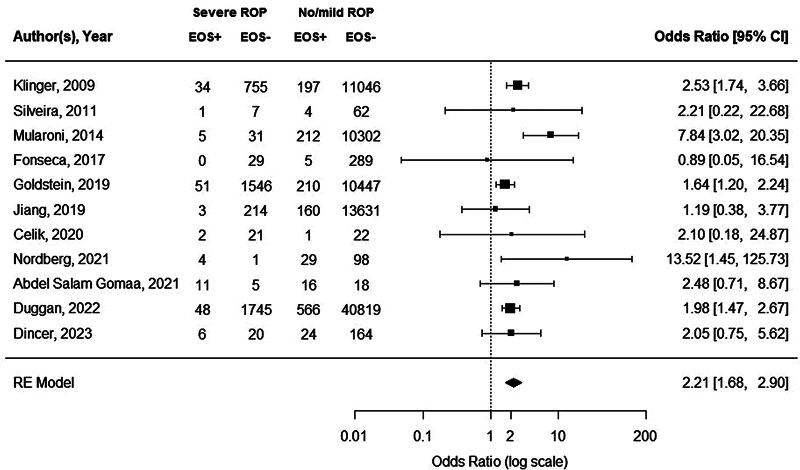
Forest plot of the association between proven EOS and severe ROP. Heterogeneity: Q = 14.8; *I* ^2^ = 32.4%; *p* = 0.1395. 95% CI, 95% confidence interval; EOS, early-onset sepsis; ROP, retinopathy of prematurity.

Eleven studies assessed the presence of proven EOS in neonates with severe ROP compared with neonates with no/mild ROP, with sample sizes ranging from 46 to 43,178 ( Supplementary Table S2 [available in the online version]). 25 27 28 29 34 35 36 37 38 39 40 Proven EOS was detected twice as frequently in neonates with severe ROP compared with neonates with no/mild ROP (OR = 2.21; 95% CI: 1.68–2.90, *p* < 0.0001; Fig. 3 ). Moderate heterogeneity was observed (Q = 14.8, *I* ^2^ = 32.4%, *p* = 0.1). Neither visual inspection and trim-and-fill number ( *n* = 1) of the funnel plot, nor the failsafe number ( *n* = 191, Kendall's τ = 0.02, *p* = 1.0) showed indication of publication bias ( Supplementary Fig. S2 [available in the online version]).

In univariate meta-regression analysis ( Table 3 ), no factors were found to be significant moderators for the association between proven EOS and severe ROP (number of studies: 3–6).

**Table 3:** Meta-regression regarding the influence of potential confounders on early-onset sepsis and severe retinopathy of prematurity

Univariate	*N*	Estimate	SE	Z-value	*p* -Value	CI lower	CI higher	*R*^2^ (%)	*I*^2^ (%)
Gestational age at birth	6	0.0193	0.1427	0.1351	0.8925	-0.2605	0.2990	0.00	0.00
Birth weight	6	−0.0001	0.0011	−0.0664	0.9470	−0.0023	0.0021	0.00	0.00
Small for gestational age	3	0.0040	0.0131	0.3059	0.7597	−0.0216	0.0216	0.00	0.00
Maternal steroids	4	0.0202	0.0332	0.6089	0.5426	−0.0448	0.0853	0.00	0.00
Necrotizing enterocolitis	5	−0.0248	0.0521	−0.4751	0.6347	−0.1269	0.0774	0.00	0.00
Late-onset sepsis	5	−0.0194	0.0315	−0.6155	0.5383	−0.0812	0.0424	0.00	0.00
Mechanical ventilation	3	−0.0128	0.0231	−0.5568	0.5776	−0.0580	0.0323	0.00	0.00

Abbreviation: CI, confidence interval.

## Discussion

This is the first meta-analysis investigating the effect of EOS on ROP, which shows that neonates with EOS have a 2.2-fold increased risk of severe ROP. These findings are based on neonates with proven EOS (i.e., positive blood or cerebrospinal fluid culture), which ensured high validity and robust evidence. Furthermore, no significant heterogeneity was observed between studies, and no significant confounders influencing this effect size were found in meta-regression analysis.

The underlying pathophysiological mechanism behind the increased ROP risk in neonates with EOS is yet to be resolved but is believed to be due to the stimulating effect of EOS on inflammatory mediators. 41 Perinatal infection leads to decreased IGF-1 levels, which promotes phase I of ROP. 12 13 42 Proinflammatory cytokines such as interleukin-1β and tumor necrosis factor-α increase HIF-1α levels. 9 The HIF-1α pathway regulates VEGF-2 production and increased HIF-1α levels may, therefore, exacerbate phase II of ROP. 43 Furthermore, severe ROP has been found to be related to increased cytokine levels in the first 72 hours of life. 44 45

These pathophysiological pathways are similar in neonates with histological chorioamnionitis, which is an acute maternal inflammatory response in the placental membranes mainly due to ascending microorganisms. 46 47 This maternal response can be accompanied by funisitis, which is the fetal inflammatory response in the umbilical cord vessels. Our previous meta-analysis has shown that neonates with placental inflammation have an increased risk for ROP and that this risk is greater when inflammation is present in the umbilical cord (funisitis). 16 EOS is also acquired through vertical transmission and can develop intrauterine as a consequence of placental inflammation.

Besides placental inflammation, LOS has also been found to increase the risk of ROP. 48 49 LOS is hospital- and community-acquired beyond the first 72 hours of life and can induce an oxidative stress response, which promotes vascular endothelial cell proliferation and migration through VEGF-2. 11 Elevated VEGF-2 levels, in response to hypoxia, cause neovascular proliferation and may therefore initiate phase II of ROP. 12 In our meta-regression analysis, LOS did not appear to influence the effect size between EOS and severe ROP. Multiple hits of antenatal/postnatal inflammation have been reported to increase the risk of ROP. 50 51 Remarkably, a recent review by Dammann and Stansfield has provided evidence that neonatal sepsis is a causal initiator of ROP and that placental inflammation can be considered a causal facilitator, which increases the likelihood of ROP. 52 Taken together, neonates with EOS have an increased risk of ROP and this risk is intensified when other inflammatory morbidities are present.

A previous meta-analysis by Wang et al explored the association between sepsis and ROP and also found that sepsis was significantly associated with severe ROP (OR = 2.3). 48 However, no distinction was made between EOS and LOS and most sepsis cases were LOS. Additionally, only six studies were included in the analysis of severe ROP (defined as ≥stage 3). Furthermore, extracted outcome data were based on adjusted ORs with most studies controlling for both GA and BW, which may have introduced multicollinearity and, thus, provided limited evidence.

Huang et al also performed a meta-analysis to identify the impact of EOS and LOS on ROP and showed similar results for severe ROP (OR = 1.9). 49 Similar to our study, Huang et al showed in subgroup analysis that EOS was significantly associated with severe ROP (OR = 2.5). However, it is important to note that Huang et al only included two studies in this analysis, which is insufficient to draw conclusions. Additionally, extracted outcome data were again based on adjusted ORs, and many studies adjusted for both GA and BW in multivariate analysis. Our meta-analysis included a relatively larger number of studies ( *n* = 10 any stage ROP, *n* = 11 severe ROP) published between 2009 and 2024 and, thus, provides stronger evidence.

Instead of exploring the overall effect of suspected/proven EOS on ROP, our study only investigated the independent effect of proven EOS on ROP to provide robust results. Remarkably, studies have shown that over 95% of neonates treated with antibiotics for suspected infection ultimately do not have sepsis. 14 Most studies do not distinguish between proven and suspected sepsis, even though it leads to overestimating the effect of sepsis on ROP. Hence, diagnosing EOS based on a positive blood or cerebrospinal fluid culture is more reliable and should be implemented in future studies.

## Limitations

The main limitation of this study was the retrospective designs of most included studies, which may have resulted in information bias. Additionally, high heterogeneity was present between included studies in the any stage ROP group, which may have affected our results. Furthermore, data on potential confounders in neonates with EOS were limited and hampered multivariate meta-regression analysis. Nevertheless, our meta-analysis provides an extensive overview based on a relatively large number of studies and is the first to explore the independent effect of proven EOS on ROP. Additionally, we eliminated heterogeneity in confounder adjustment by calculating unadjusted ORs from 2 × 2 tables reported in the included studies.

## Conclusion

In conclusion, this meta-analysis introduces proven EOS as an early risk factor for severe ROP. Future studies should diagnose sepsis based on positive blood or cerebrospinal fluid cultures and distinguish between proven EOS and LOS to provide reliable and stronger evidence. Neonatologists need to be aware that EOS is an early predictor of ROP that should be taken into account in neonatal policies and treatment decisions well before ROP screening begins.
