# Supplementary material for: Early-Onset Sepsis as an Early Predictor for Retinopathy of Prematurity: A Meta-analysis
Source: Am J Perinatol. 2024 Aug 13;42(3):387–94. doi: 10.1055/a-2369-6690 (PMC11793952; doi:10.1055/a-2369-6690)
Supplement: Supplementary file 1 — Supplementary Material [file 10-1055-a-2369-6690-s24apr0214.pdf]

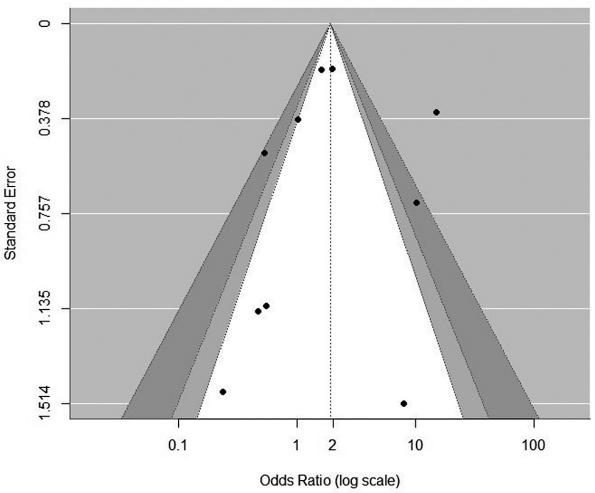

Supplementary Fig. S1 Funnel plot for publication bias assessment of studies reporting on proven EOS and any stage ROP. EOS, early-onset sepsis; ROP, retinopathy of prematurity.

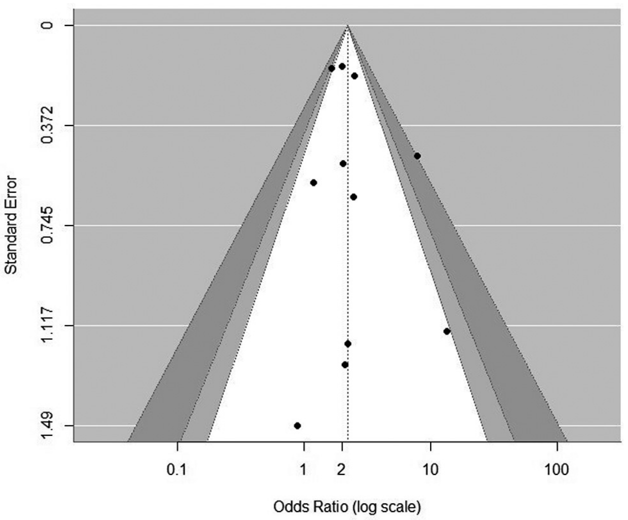

Supplementary Fig. S2 Funnel plot for publication bias assessment of studies reporting on proven EOS and severe ROP. EOS, early-onset sepsis; ROP, retinopathy of prematurity.

Supplementary Material S1 PRISMA 2020 Checklist

Section and topic	Item number	Checklist item	Location where the item is reported
**Title**			
Title	1	Identify the report as a systematic review	1
**Abstract**			
Abstract	2	See the PRISMA 2020 for the Abstracts checklist	2
**Introduction**			
Rationale	3	Describe the rationale for the review in the context of existing knowledge	4
Objectives	4	Provide an explicit statement of the objective(s) or question (s) the review addresses	4, 5
**Methods**			
Eligibility criteria	5	Specify the inclusion and exclusion criteria for the review and how studies were grouped for the syntheses	5, 6
Information sources	6	Specify all databases, registers, websites, organizations, reference lists, and other sources searched or consulted to identify studies. Specify the date when each source was last searched or consulted	5

(*Continued*)

(*Continued*)

Section and topic	Item number	Checklist item	Location where the item is reported
Search strategy	7	Present the full search strategies for all databases, registers, and websites, including any filters and limits used	5
Selection process	8	Specify the methods used to decide whether a study met the inclusion criteria of the review, including how many reviewers screened each record and each report retrieved, whether they worked independently, and if applicable, details of automation tools used in the process	5
Data collection process	9	Specify the methods used to collect data from reports, including how many reviewers collected data from each report, whether they worked independently, any processes for obtaining or confirming data from study investigators, and if applicable, details of automation tools used in the process	5
Data items	10a	List and define all outcomes for which data were sought. Specify whether all results that were compatible with each outcome domain in each study were sought (e.g., for all measures, time points, analyses), and if not, the methods used to decide which results to collect	5
	10b	List and define all other variables for which data were sought (e.g., participant and intervention characteristics, funding sources). Describe any assumptions made about any missing or unclear information	5, 6
Study the risk of bias assessment	11	Specify the methods used to assess the risk of bias in the included studies, including details of the tool(s) used, how many reviewers assessed each study whether they worked independently, and if applicable, details of automation tools used in the process	5, 6
Effect measures	12	Specify for each outcome the effect measure(s) (e.g., risk ratio, mean difference) used in the synthesis or presentation of results	6, 7
Synthesis methods	13a	Describe the processes used to decide which studies were eligible for each synthesis (e.g., tabulating the study intervention characteristics and comparing against the planned groups for each synthesis [item number 5]).	5, 6
	13b	Describe any methods required to prepare the data for presentation or synthesis, such as handling missing summary statistics, or data conversions	6
	13c	Describe any methods used to tabulate or visually display the results of individual studies and syntheses	6
	13d		6, 7

(*Continued*)

Section and topic	Item number	Checklist item	Location where the item is reported
		Describe any methods used to synthesize results and provide a rationale for the choice(s). If meta-analysis was performed, describe the model(s), method(s) to identify the presence and extent of statistical heterogeneity, and software package(s) used	
	13e	Describe any methods used to explore possible causes of heterogeneity among study results (e.g., subgroup analysis, meta-regression)	6, 7
	13f	Describe any sensitivity analyses conducted to assess the robustness of the synthesized results.	6, 7
Reporting bias assessment	14	Describe any methods used to assess the risk of bias due to missing results in a synthesis (arising from reporting biases)	6, 7
Certainty assessment	15	Describe any methods used to assess certainty (or confidence) in the body of evidence for an outcome	6, 7
**Results**			
Study selection	16a	Describe the results of the search and selection process, from the number of records identified in the search to the number of studies included in the review, ideally using a flow diagram	8, flow diagram
	16b	Cite studies that might appear to meet the inclusion criteria, but which were excluded, and explain why they were excluded	Flow diagram
Study characteristics	17	Cite each included study and present its characteristics	8, 9, and tables
Risk of bias in studies	18	Present assessments of risk of bias for each included study	8, 9, and tables
Results of individual studies	19	For all outcomes, present, for each study: (a) summary statistics for each group (where appropriate) and (b) an effect estimate and its precision (e.g., confidence/credible interval), ideally using structured tables or plots	Tables and forest plot
Results of syntheses	20a	For each synthesis, briefly summarize the characteristics and risk of bias among contributing studies	8, 9
	20b	Present results of all statistical syntheses conducted. If meta-analysis was done, present for each the summary estimate and its precision (e.g., confidence/credible interval) and measures of statistical heterogeneity. If comparing groups, describe the direction of the effect	8, 9
	20c		8, 9

(*Continued*)

(*Continued*)

Section and topic	Item number	Checklist item	Location where the item is reported
		Present results of all investigations of possible causes of heterogeneity among study results	
	20d	Present results of all sensitivity analyses conducted to assess the robustness of the synthesized results	8, 9
Reporting biases	21	Present assessments of risk of bias due to missing results (arising from reporting biases) for each synthesis assessed	8, 9
Certainty of evidence	22	Present assessments of certainty (or confidence) in the body of evidence for each outcome assessed	8, 9
**Discussion**			
Discussion	23a	Provide a general interpretation of the results in the context of other evidence	9–11
	23b	Discuss any limitations of the evidence included in the review	11, 12
	23c	Discuss any limitations of the review processes used	12
	23d	Discuss the implications of the results for practice, policy, and future research	12
**Other Information**			
Registration and protocol	24a	Provide registration information for the review, including the register name and registration number, or state that the review was not registered	5
	24b	Indicate where the review protocol can be accessed, or state that a protocol was not prepared	5
	24c	Describe and explain any amendments to information provided at registration or in the protocol	N/A
Support	25	Describe sources of financial or non-financial support for the review, and the role of the funders or sponsors in the review	13
Competing interests	26	Declare any competing interests of review authors	13
Availability of data, code, and other materials	27	Report which of the following are publicly available and where they can be found: template data collection forms; data extracted from included studies; data used for all analyses; analytic code; any other materials used in the review	N/A

Modified from: Page MJ, McKenzie JE, Bossuyt PM, Boutron I, Hoffmann TC, Mulrow CD, et al. The PRISMA 2020 statement: an updated guideline for reporting systematic reviews. BMJ 2021;372:n71. doi: 10.1136/bmj.n71. For more information, visit: http://www.prisma-statement.org/

 © 2024. The Author(s).

Supplementary Table S1 Study characteristics of any stage retinopathy of prematurity with early-onset sepsis

First author, year	Study design	Population	Patients	Definition of sepsis	Diagnosis of ROP	Outcome	Validity
Chen, 2011[24]	Retrospective cohort study	The United States, 1 997–2007	EOS: $n = 3$<br>No EOS: $n = 619$	Recovered bacterial pathogen from a blood and/or cerebrospinal fluid culture obtained on first 3 days of life	ROP screening was performed by two attending retinal specialists	EOS<br>● Any stage ROP: 3/293 (1)<br>● No ROP: 0/329 (0)	8 points
Silveira, 2011[25]	Prospective cohort study	Brazil, 2005–2007	EOS: $n = 5$<br>No EOS: $n = 69$	Positive blood cultures <72 hours after birth	ROP screening was performed by the same ophthalmologist through binocular indirect ophthalmoscopy	EOS ($p = 0.657$)<br>● Any stage ROP: 1/25 (4)<br>● No ROP: 4/49 (8)	7 points
Jimenez, 2012[26]	Retrospective case-control study	Mexico, 2004–2007	EOS: $n = 43$<br>No EOS: $n = 74$	Clinical and hematological evidence of sepsis in the first 72 hours of life	ROP screening inclusion was based on the American Academy of Pediatrics and Ophthalmology guidelines, which recommend using the ICROP for ROP staging	EOS<br>● Any stage ROP: 21/57 (37)<br>● No ROP: 22/60 (37)	8 points
Fonseca, 2018[27]	Prospective/ Retrospective cohort study	Brazil, 2011–2014	EOS: $n = 5$<br>No EOS: $n = 318$	Confirmed by blood culture	ROP screening was performed by a single trained ophthalmologist through retina mapping tests	EOS ($p = 0.635$)<br>● Any stage ROP: 1/100 (1)<br>● No ROP: 4/223 (2)	9 points
Jiang, 2019[28]	Prospective cohort study	China, 2015–2018	EOS: $n = 163$<br>No EOS: $n = 13{,}845$	Presence of clinical symptoms and a positive culture from blood or cerebrospinal fluid drawn within 72 hours of birth	ICROP2 was used for ROP staging	EOS<br>● Any stage ROP: 40/2,382 (2)<br>● No ROP: 123/11,626 (1)	8 points
Nordberg, 2021[29]	Retrospective cohort study	Sweden, 2006–2016	EOS: $n = 33$<br>No EOS: $n = 99$	Positive gram-negative bacteria blood culture before 72 hours of age	ROP screening was based on Swedish national guidelines, which recommend that ROP screening is performed by an experienced pediatric ophthalmologist through fundus photography with wide-angle technology or, if necessary, indirect ophthalmoscopy	EOS<br>● Any stage ROP: 8/11 (73)<br>● No ROP: 25/121 (21)	7 points
Carranza-Mendizabal, 2021[30]	Retrospective cohort study	Peru, 2016–2018	EOS: $n = 78$<br>No EOS: $n = 138$	Positive bacterial culture in blood or	ROP screening was performed by the ophthalmology specialist	EOS ($p < 0.001$)<br>● Any stage ROP: 54/72 (75)	8 points

(*Continued*)

Supplementary Table S1 (*Continued*)

First author, year	Study design	Population	Patients	Definition of sepsis	Diagnosis of ROP	Outcome	Validity
				cerebrospinal fluid at <72 hours of age	through indirect ophthalmoscopy	● No ROP: 24/144 (16.7)	
Bonafiglia, 2022[31]	Retrospective cohort study	Italy, 2015–2019	EOS: $n=7$<br>No EOS: $n=273$	Isolated bacterial pathogen from blood and/or cerebrospinal fluid culture before the first 3 days of life	ROP screening was performed by two board-certified consultant ophthalmologists through binocular indirect ophthalmoscopy and confirmed by fundus photography. ICROP was used for ROP staging	EOS ($p=0.16$)<br>● Any stage ROP: 0/60 (0)<br>● No ROP: 7/220 (3)	8 points
Gudu, 2022[32]	Retrospective cohort study	India, 2019	EOS: $n=28$<br>No EOS: $n=57$	Clinical signs and screening markers (i.e., total leukocyte count, absolute neutrophil count, and C-reactive protein) along with positive blood culture	ROP screening was performed by a trained ophthalmologist under the supervision of a neonatologist through indirect ophthalmoscopy	EOS ($p=0.217$)<br>● Any stage ROP: 7/29 (24)<br>● No ROP: 21/56 (38)	7 points
Boo, 2024[33]	Retrospective cohort study	Malaysia, 2015–2020	EOS: $n=161$<br>No EOS: $n=10{,}275$	Unwell neonates with positive blood culture at ≤72 hours of life	ROP screening was performed by ophthalmologists while using the Malaysian national guidelines, which recommend using binocular indirect ophthalmoscopy and, if severe, wide-field retinal imaging	EOS ($p<0.001$)<br>● Any stage ROP: 44/1,685 (3)<br>● No ROP: 117/8,751 (1)	9 points

Abbreviations: EOS, early-onset sepsis; ICROP, International Classification of retinopathy of prematurity; ROP, retinopathy of prematurity.
Data are presented as *n*/*N* (%).

 © 2024. The Author(s).

Supplementary Table S2 Study characteristics of severe retinopathy of prematurity with early-onset sepsis

First author, year	Study design	Population	Patients	Definition of sepsis	Diagnosis of ROP	Outcome	Validity
Klinger, 2010[34]	Prospective cohort study	Israel, 1995–2005	EOS: $n = 231$<br>No EOS: $n = 11,801$	Defined clinically and required a positive blood culture obtained within the first 72 hours of life	ROP was determined and graded by ophthalmologic examination according to the ICROP	EOS<br>● Severe ROP: 34/789 (4)<br>● No/mild ROP: 197/11,243 (2)	9 points
Silveira, 2011[25]	Prospective cohort study	Brazil, 2005–2007	EOS: $n = 5$<br>No EOS: $n = 69$	Positive blood cultures <72 hours after birth	ROP screening was performed by the same ophthalmologist through binocular indirect ophthalmoscopy	EOS ($p = 0.445$)<br>● Severe ROP: 1/8 (13)<br>● No/mild ROP: 4/66 (6)	7 points
Mularoni, 2014[35]	Prospective cohort study	Spain, 2006–2009	EOS: $n = 391$<br>No EOS: $n = 14,328$	Presence of clinical symptoms and a positive blood culture drawn within 72 hours of birth	ROP data were extracted from the European Neonatal Network database, which included data from 200 European NICUs. ROP was diagnosed according to their standardized definitions but they cannot be accessed anymore	EOS ($p < 0.001$)<br>● Severe ROP: 5/36 (14)<br>● No/mild ROP: 386/14,683 (3)	7 points
Fonseca, 2018[27]	Prospective/ Retrospective cohort study	Brazil, 2011–2014	EOS: $n = 5$<br>No EOS: $n = 318$	Confirmed by blood culture	ROP screening was performed by a single trained ophthalmologist through retina mapping tests	EOS ($p = 1.000$)<br>● Severe ROP: 0/29 (0)<br>● No/mild ROP: 5/294 (2)	9 points
Goldstein, 2019[36]	Prospective cohort study	United States, 2007–2011	EOS: $n = 261$<br>No EOS: $n = 11,993$	Bacterial pathogen recovered from a blood and/or cerebrospinal fluid culture obtained on day 1, 2, or 3 of life	ROP screening inclusion was based on the American Academy of Pediatrics and Ophthalmology guidelines, which recommend using the ICROP for ROP staging	EOS<br>● Severe ROP: 51/1,597 (3)<br>● No/mild ROP: 210/10,657 (2)	8 points
Jiang, 2019[28]	Prospective cohort study	China, 2015–2018	EOS: $n = 163$<br>No EOS: $n = 13,845$	Presence of clinical symptoms and a positive culture from blood or cerebrospinal fluid drawn within 72 hours of birth	ICROP2 was used for ROP staging	EOS<br>● Severe ROP: 3/217 (1)<br>● No/mild ROP: 160/13,791 (1)	8 points
Celik, 2021[37]	Retrospective cohort study	Turkey, 2018–2019	EOS: $n = 3$<br>No EOS: $n = 43$	Positive blood culture accompanied by systemic signs of infection	ROP screening was performed by an ophthalmologist who is “competent and	EOS ($p = 0.9$)<br>● Severe ROP: 2/23 (9)	7 points

(*Continued*)

 © 2024. The Author(s).

Supplementary Table S2 (*Continued*)

First author, year	Study design	Population	Patients	Definition of sepsis	Diagnosis of ROP	Outcome	Validity
				within the first 3 days of life	qualified in ROP" with a binocular indirect ophthalmoscope	● No/mild ROP: 1/23 (4)	
Nordberg, 2021[29]	Retrospective cohort study	Sweden, 2006–2016	EOS: $n = 33$<br>No EOS: $n = 99$	Positive gram-negative bacteria blood culture before 72 hours of age	ROP screening was based on Swedish national guidelines, which recommend that ROP screening is performed by an experienced pediatric ophthalmologist through fundus photography with wide-angle technology or, if necessary, indirect ophthalmoscopy	EOS ($p = 0.01$)<br>● Severe ROP: 4/5 (80)<br>● No/mild ROP: 29/127 (23)	7 points
Abdel Salam Gomaa, 2021[38]	Retrospective case–control study	Egypt, 2018–2020	EOS: $n = 27$<br>No EOS: $n = 23$	Positive blood culture or positive C-reactive protein and immature to total neutrophil ratio >0.2 with clinical signs of sepsis in the first 3 postnatal days	ROP screening was performed by a trained neonatologist through wide-field digital retinal imaging and, if detected, diagnosis was confirmed by a pediatric ophthalmologist trained in ROP through indirect ophthalmoscopy	EOS ($p = 0.151$)<br>● Severe stage ROP: 11/16 (69)<br>● Mild ROP: 16/34 (47)	7 points
Duggan, 2023[39]	Retrospective cohort study	Australia, 2007–2018	EOS: $n = 614$<br>No EOS: $n = 42,564$	Culture-positive systemic bacterial or fungal infection up to 48 hours after birth	ROP data was extracted from the Australian and New Zealand Neonatal Network database, which recommends the ICROP for ROP classification and staging	EOS<br>● Severe ROP: 48/1,793 (3)<br>● No/mild ROP: 566/41,385 (1)	9 points
Dincer, 2023[40]	Retrospective cohort study	Turkey, 2017–2018	EOS: $n = 30$<br>No EOS: $n = 184$	Positive blood culture	ROP screening was performed by an experienced ophthalmologist specializing in neonatal ophthalmology through indirect ophthalmoscope	EOS ($p = 0.133$)<br>● Severe ROP: 6/26 (23)<br>● No/mild ROP: 24/188 (13)	7 points

Abbreviations: EOS, early-onset sepsis; ICROP, international classification of retinopathy of prematurity; ROP, retinopathy of prematurity.
Data are presented as n/N (%).
